# CD146 expression on mesenchymal stem cells is associated with their vascular smooth muscle commitment

**DOI:** 10.1111/jcmm.12168

**Published:** 2013-11-04

**Authors:** Nicolas Espagnolle, Fabien Guilloton, Frédéric Deschaseaux, Mélanie Gadelorge, Luc Sensébé, Philippe Bourin

**Affiliations:** a EFS Pyrénées Méditerranée UMR5273 CNRS/UPS/EFS, Inserm U1031 STROMALabToulouse, France; b CSA21 7 chemin des silos 31100Toulouse, France

**Keywords:** CD146, vascular smooth muscle cell, mesenchymal stem cells, proliferation, differentiation

## Abstract

Bone marrow mesenchymal stem cells (MSCs) are plastic adherent cells that can differentiate into various tissue lineages, including osteoblasts, adipocytes and chondrocytes. However, this progenitor property is not shared by all cells within the MSC population. In addition, MSCs vary in their proliferation capacity and expression of markers. Because of heterogeneity of CD146 expression in the MSC population, we compared CD146^−/Low^ and CD146^High^ cells under clonal conditions and after sorting of the non-clonal cell population to determine whether this expression is associated with specific functions. CD146^−/Low^ and CD146^High^ bone marrow MSCs did not differ in colony-forming unit-fibroblast number, osteogenic, adipogenic and chondrogenic differentiation or *in vitro* haematopoietic-supportive activity. However, CD146^−/Low^ clones proliferated slightly but significantly faster than did CD146^High^ clones. In addition, a strong expression of CD146 molecule was associated with a commitment to a vascular smooth muscle cell (VSMC) lineage characterized by a strong up-regulation of calponin-1 and SM22α expression and an ability to contract collagen matrix. Thus, within a bone marrow MSC population, certain subpopulations characterized by high expression of CD146, are committed towards a VSMC lineage.

## Introduction

Mesenchymal stem/stromal cells (MSC) are multipotent progenitors that give rise to skeletal cells (osteoblasts, chondrocytes, haematopoietic-supportive stromal cells) and adipocytes 1–4. They are relatively easy to expand, and their progenitor nature is evaluated by colony-forming unit-fibroblast (CFU-F) assay 5. Although MSCs are often characterized after a period of culture, recently, many studies have aimed to define their native form in bone marrow aspirates. These works emphasize different membrane molecules as native MSC markers. Among them, CD200, a marker for MSCs appeared to be a good marker to reproducibly purify native MSCs. The *in vitro* adipogenic, osteogenic and chondrogenic potential of CD200^+^ cells was similar to that for cells separated by adherence 6. Sacchetti *et al*., from cultured and native cells by using sorting and clonal methods, found CD146 as a key marker for bone marrow MSCs (BM-MSCs). Culture-expanded clonogenic CD146+ cells and not CD146− cells were osteogenic *in vivo* and could re-establish the haematopoietic microenvironment in a xenotransplantation model 7. A homogenous CD45−/CD146+ multipotent MSC population deriving from human BM exhibited haematopoiesis-supporting abilities, an extensive 12-week proliferation and the ability to differentiate in osteoblasts, chondrocytes and adipocytes 8. Another study suggested that CD146 might be a marker of clonal multipotent cultured MSCs 9.

However, from CD271+/CD45− BM fractions Tormin *et al*. observed CD146−/low and CD146+ populations with CFU-F potential with any differences in multilineage differentiation capacity. Moreover, the CD146 expression by BM-MSCs depended on the environment: up-regulated under normoxia and down-regulated under hypoxia. This O_2_-related expression was associated with the *in situ* localization: CD146 expressing reticular cells were located in perivascular regions, whereas cells close to the bone surface were CD146− 10. In addition, adipose-tissue perivascular cells with MSC properties, such as pericytes of microvessels and capillaries, were identified to have high CD146 expression, whereas other cells from tunica adventitia from large vessels lacked CD146 11–12. Therefore, the MSC expression of CD146 is heterogeneous and may depend on the tissue and the molecular environment. This observation suggests functional differences.

Therefore, in this study, we investigated BM-MSC functions in terms of CD146 expression. We compared sorted and clonogenic CD146^−/Low^ and CD146^High^ cells after *in vitro* expansion and examined the different properties of MSC such as osteogenic, chondrogenic, adipogenic and vascular smooth muscle cell (VSMC) differentiation; haematopoiesis support; proliferation; CFU-F formation; transcriptome and phenotype to distinguish between these two BM-MSC subpopulations.

## Material and methods

### Preparation of single cell-derived clonal-cultured MSCs and sorted MSCs

Human BM-MSCs were isolated by culture from the BM of healthy donors obtained during the preparation of allogeneic haematopoietic stem cell grafts. This tissue is considered waste material in France and does not require informed consent for use, in accordance with French ethical and legal regulations. Briefly, BM-MSCs were obtained from unprocessed BM without red blood lysis nor density-gradient method and seeded at 5 × 10^4^ cells/cm^2^ into a 150-cm^2^ flask with minimum essential medium α (αMEM; Life Technologies, Saint Aubin, France) supplemented with 10% foetal calf serum (FCS; Lonza, Levallois-Perret, France) and 10 μg/ml ciprofloxacin (Bayer, Puteaux, France). For all MSC cultures, the medium was renewed twice a week until cells reached confluence (P1). Cells were then detached with trypsin (Gibco, Life Technologies) 13–14.

For clonal studies, total BM cells were seeded in 24-well plates at 1–2 × 10^4^/cm^2^/well in αMEM supplemented with 10% FCS and ciprofloxacin. Wells were screened every day starting at day 7 to identify wells with one clone. After 10–14 days, the wells containing only one colony (CFU-F) of MSCs were selected and cells were expanded. After expansion, MSC clones were detached by use of trypsin (Invitrogen), counted and submitted to phenotypic characterization. Cytometry was used to select clones on the basis of CD146 expression: CD146 mean fluorescence intensity was used to determine CD146^Low^ and CD146^High^ clones. The doubling population number was calculated by considering the number of CFU-Fs as the number of initiating cells at day 0.

For non-clonal studies, BM cells were seeded at 5 × 10^4^ cells/cm^2^ in the same medium at day 0 for 21 days (P1). After trypsin treatment, MSCs at passage P1 were used for phenotypic and functional studies. To sort CD146+ and CD146− MSCs, MSCs were incubated with anti-FITC-CD146 antibody (Becton-Dickinson, Le Pont de Claix, France) and separated by use of a fluorescent activated cell sorter (BD ARIA II, “Service commun de cytometrie de Toulouse Purpan”). Sorted cells were seeded at 1000 cells/cm^2^ and grown to confluence to calculate population doubling.

### Flow cytometry

Clonal BM-MSCs were phenotyped by flow cytometry with the FITC- or phycoerythrin-conjugated antibodies anti-CD90, anti-CD45, anti-CD13 (Beckman-Coulter, Villepinte, France), anti-CD73, anti-CD105 and anti-CD146 (clone P1H12) (Becton-Dickinson) and Allophycocyanin-conjugated anti-CD146 (clone 541-10B2; Miltenyi, Bergisch Gladbach, Germany) or isotype control monoclonal antibodies. Samples were analysed by use of an ADPCyan flow cytometer and Kaluza software (Beckman-Coulter).

### CFU-F assay

To evaluate the frequency of CFU-Fs generated, clonal BM-MSCs were seeded at 8 cells/cm^2^ for 10 days. Cultures were then washed with PBS, fixed with methanol and stained with Giemsa (Oxoid, Dardilly, France); colonies with more than 50 cells were counted.

### Incorporation of 5-ethynyl-2′-deoxyuridine (EdU)

During last 3 days of culture, non-clonal and clonal BM-MSCs were incubated with 5 μM Click-iT EdU (Molecular Probes, Life Technologies). After treatment, clonal BM-MSCs were stained with APC-conjugated anti-CD146 monoclonal antibody, fixed, permeabilized and treated with a Click-iT reaction cocktail for detection following the manufacturer’s recommendations. Samples were analysed by use of an ADPCyan flow cytometer and Kaluza software (Beckman-Coulter).

### VSM differentiation

Non-clonal BM-MSCs from different donors were cultured in a F25 flask with αMEM + 10% FCS + 10 μg/ml ciprofloxacin with or without 2 ng/ml fibroblast growth factor 2 (FGF-2) 15 or transforming growth factor β1 (TGF-β1; R&D Systems, Minneapolis, MN, USA) for 10 days 16–17.

### Immunofluorescence

Clonal MSCs or VSM-differentiated MSCs were fixed, permeabilized and incubated with purified primary antibodies mouse anti-human calponin-1 (anti-CNN1; Abcam, Bristol, UK), rabbit anti-human smooth muscle 22α (anti-SM22α; Abcam) or isotype control (Abcam) overnight at 4°C. After a wash with PBS, cells were incubated with secondary goat antimouse or anti-rabbit-alexa 488 (Invitrogen). Samples were analysed under a fluorescence microscope Olympus (Tokyo, Japan) IX71, ×20 objective.

### Osteogenic, adipogenic and chondrogenic differentiation

For osteogenic differentiation, cells were seeded at 5 × 10^3^ cells/cm² and differentiated for 21 days in inductive medium (αMEM + 10% FCS, dexamethasone 100 nM, ascorbic acid 2-phosphate 50 μM, sodium phosphate monobasic 3 mM/β-glycerophosphate 10 mM). Cell mineralization was then evaluated by alizarin red staining (40 mM alizarin red solution) 18. For adipogenic differentiation, cells were seeded at 2 × 10^4^ cells/cm^2^ and differentiated for 21 days in medium (αMEM, 10% FCS, dexamethasone 1 μM, 3-isobutyl-1-methylxanthine 0.45 mM, indomethacin 60 μM), then stained with 5 μg/ml Nile Red solution (lipid vacuole staining) and 2 μg/ml bisBenzimide solution (nuclear staining) 19. For both differentiations, a control test involved cells grown in αMEM with 10% FCS for 21 days. For RT-PCR analysis of *in vitro* differentiation assays, MSCs were seeded in 6-well plates (2 × 10^4^ cell/cm^2^) and allowed to reach confluence before treatment. Medium was changed twice a week. For osteoinduction, MSCs were stimulated with 50 ng/ml recombinant human bone morphogenic protein 4 (BMP4; Stemgent, Cambridge, UK) in αMEM+2% FCS for 21 days. For adipogenesis and chondrogenesis, MSCs were cultivated in adipogenic differentiation medium (Miltenyi Biotech, Bergisch Gladbach, France) for 10 days and chondrogenic differentiation medium (Miltenyi Biotech) + 10 ng/ml recombinant human TGF-β3 (R&D Systems) for 21 days.

### Co-culture of clonal BM-MSCs and CD34+ cells

CD146^−/Low^ and CD146^High^ clonal BM-MSCs were seeded in 12-well plates in duplicate at 2 × 10^4^ cells/well in 1 ml culture medium. After 24 hrs, BM-MSCs were co-cultured with 4 × 10^4^ CD34^+^ cells in 2 ml Myelocult medium containing 10 μM hydrocortisone (Stem Cell Technologies, Grenoble, France). At days 7, 14, 21 and 28, non-adherent cells were collected, counted and assayed for haematopoietic progenitor content by use of the methyl-cellulose semisolid culture type (Miltenyi Biotec). The total number of clonogenic progenitors was calculated (number of colonies/number of seeded cells in methyl-cellulose) × total number of cells in the co-culture). Colony-forming unit-granulocytes (Gs), CFU-macrophages (Ms), CFU-granulocyte macrophages (GMs) and burst-forming unit-erythroids (BFU-Es) were counted.

### Affymetrix microarrays

After clonal BM-MSC culture and CD146 expression analysis, the RNA in non-clonal and clonal CD146^−/Low^ and CD146^High^ MSCs from four donors was extracted by use of the RNeasy Kit (Qiagen, Hilden, Germany). RNA purity and integrity were checked by use of Bioanalyzer 2100 (Agilent, Santa Clara, CA, USA). The RNA integrity number was >9 for all samples used for microarray assays. Biotinylated cRNA was amplified by the small sample labelling protocol (TwoCycle amplification kit, Affymetrix, Santa Clara, CA, USA) and hybridized on GeneChip Hugene 1.0 ST oligonucleotide microarrays (Affymetrix). Expression signal values and *P*-values were obtained for each probe set by use of Partek Genomics Suite (Partek, St. Louis, MO, USA) by the Robust Multichip Averaging algorithm in normalization. Principal component and gene expression analysis involved use of MeV.

The accession number in the Geo data set in Medline is available at http://www.ncbi.nlm.nih.gov/geo/query/acc.cgi?acc=GSE48540.

### RNA extraction/cDNA synthesis and quantitative PCR (qPCR)

RNA was extracted by use of the AllPrep ARN/ADN/protein kit (Qiagen, Valencia, CA, USA). cDNA synthesis was performed with 1 μg total RNA with the High Capacity cDNA reverse-transcription kit (Applied Biosystems, Life Technologies) and random hexamers. qPCR was performed on diluted cDNA (equivalent to 25 ng of starting purified RNA) with SSoFast EvaGreen Supermix (Biorad) and 500 nM forward and reverse primers in a total volume of 20 μL on a CFX Real Time System (Bio-Rad, Marnes-la-Coquette, France) at 95°C, 3 min. and 40 cycles of denaturation (95°C, 10 sec.) and primer hybridation and amplification (60°C, 30 sec.). Primer sequences are in Table 1. Each primer couple displayed interpretable PCR efficiencies (95–105%). Melting curves and an appropriate No-RT control were used to validate amplification specificity. Data were analysed by use of the Bio-Rad CFX manager (threshold=0.2) and exported to DataAssist Software v3.0 (Applied Biosystems). Gene expression was calculated by the 2^−ΔCt^ (or 2^−ΔΔCt^) method with PPIA used as an appropriate reference gene because it had the lesser M score.

**Table 1:** List of primers

	Forward primer	Reverse primer
ALPL	5′-CCTGGAGCTTCAGAAGCTCAA-3′	5′-ACTGTG GAGACACCCATCCC-3′
DLX5	5′-GCCACCAACCAGCCAGAGAA-3′	5′-GCGAGGTACTGAGTCTTCTGAAACC-3′
RUNX2	5′-GGCCCACAAATCTCAGATCGTT-3′	5′-CACTGGCGCTGCAACAAGAC-3′
OSX	5′-CTCCTGCGACTGCCCTAAT-3′	5′-GCCTTGCCATACACCTTGC-3′
PPARγ2	5′-GATACACTGTCTGCAAACATATCAC-3′	5′-CCACGGAGCTGATCCCAA-3′
FABP4	5′-AGTGAAAACTTTGATGATTATATG-3′	5′-CCATGCCAGCCACTTTCCT-3′
COL10A1	5′-GGTATAGCAGTAAGAGGAGAGCA-3′	5′-AGGACTTCCGTAGCCTGGTTT-3′
CNN1	5′-GCATGTCCTCTGCTCACTTCAA-3′	5′-GGGCCAGCTTGTTCTTAACCT-3′
SM22A	5′-TTGGATCCGACATGGCCAACAAG-3′	5′-AGATCATCAGTTAGAAAGCTTAGGGC-3′
NANOG	5′-TGGACACTGGCTGAATCCTTC-3′	5′-CGTTGATTAGGCTCCAACCAT-3′
SOX2	5′-CCATCCACACTCACGCAAAA-3′	5′-CCCCCAAAAAGAAGTCCCAA-3′
OCT4A	5′-AGTGAGAGGCAACCTGGAGA-3′	5′-GTGAAGTGAGGGCTCCCATA-3′
ELN	5′-AACCAGCCTTGCCCGC-3′	5′-CCCCAAGCTGCCTGGTG-3′

### Cell contraction assay

CD146^High/Low^ clones were incubated in collagen matrices in 24-well plates for 24 hrs during which stress developed. To initiate contraction, we released collagen gels from the sides of the culture dishes. After 5 hrs, the surface area of the matrix contracted was expressed as a percentage of its initial surface area.

### Statistical analysis

Data are expressed as mean ± SEM of (*n*) separate experiments. Statistical comparisons involved Student’s *t*-test and GraphPad Prism software (La Jolla, CA, USA). Differences were considered statistically significant at *P* < 0.05.

## Results

### CFU-F and proliferation potential of MSCs and CD146 expression

**Figure 1:**
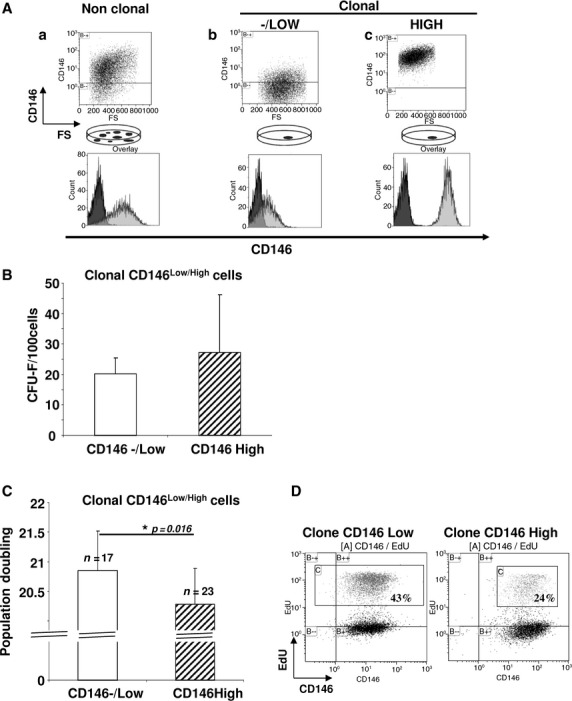
Mesenchymal stem cells (MSC) proliferation is higher with CD146^−/Low^ than CD146^High^ expression. (A) Flow cytometry of CD146 expression in MSCs derived from non-clonal (a) or clonal (b-c) conditions. Several clones were analysed for CD146 expression, and one CD146^−/Low^ clone and one CD146^High^ clone among 20 clones are shown. (B) Colony-forming unit-fibroblast (CFU-F) number in clones seeded at 8 cells/cm^2^ in F25 flasks at 10 days. Data are mean ± SEM CFU-Fs for 100 seeded cells (*n* = 6). (C) Population doubling number calculated as Log^2^ (cell expansion) in CD146^−/Low^ and CD146^High^ clonal MSCs expanded for 4 weeks (*n* = 17 for CD146^−/Low^ and *n* = 23 for CD146^High^). Data are mean ± SEM. (D) Flow cytometry of CD146 population with 5-ethynyl-2′-deoxyuridine (EdU) incorporation in CD146^−/Low^ and CD146^High^ clonal MSCs for 3 days. Data are representative of three independent experiments.

From BM, we expanded non-clonal MSCs or clonal MSCs and selected them by CD146 expression (CD146^−/Low^ and CD146^High^, Fig. 1A). For non-clonal cells, CD146 expression was heterogeneous (Fig. 1Aa), but clonogenic cells showed homogenous negative-to-low or high expression of CD146 (CD146^−/Low^ and CD146^High^) (Fig. 1Ab and c). Clonal CD146^−/Low^ and CD146^High^ fractions produced a similar number of CFU-Fs (20 ± 5 *versus* 27 ± 19, respectively; Fig. 1B). In addition, sorted non-clonal CD146^−/Low^ and CD146^High^ cells did not differ in generating CFU-Fs (Fig. S1A and B).

Proliferation potential was slightly but significantly higher for clonal CD146^−/Low^ than CD146^High^ cells (population doubling number: 20.9 ± 0.7 *versus* 20.3 ± 0.6, *P* = 0.016) at week 4 (Fig. 1C) and was similar with sorted non-clonal CD146−/+ cells (Fig. S1C). As confirmed by flow cytometry, the proportion of cycling cells (percentage of EdU-incorporated cells) was significantly higher in the clonal CD146^−/Low^ than CD146^High^ fraction (43% *versus* 24%, Fig. 1D). Of note, CD146 expression was enhanced in clonal CD146^−/Low^ cells after proliferation period but did not reach the level detected in the clonal CD146^High^ population.

### Functional assessment of CD146^−/Low^ and CD146^High^ MSCs

**Figure 2:**
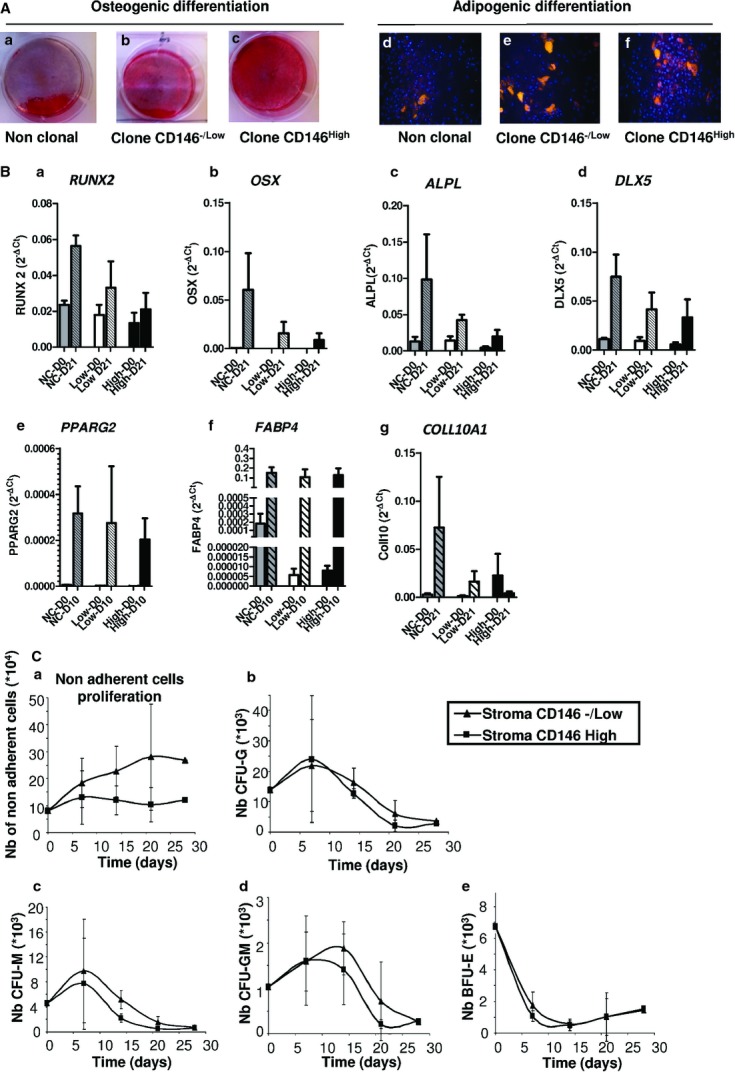
CD146^−/Low^ and CD146^High^ cells show similar mesenchymal stem cells (MSC) characteristics. (A) Osteogenic or adipogenic differentiation of non-clonal and CD146^Low/High^ clonal MSCs stained with alizarine red (a–c) or Nile red (d–f). Images are representative of three independent experiments. (B) Quantitative RT-PCR and transcriptome analysis of osteoblast (RUNX2, OSX, ALPL and DLX5), adipocyte (PPARγ2, FABP4) and chondroblast (COLL10A1) marker genes in non-clonal (NC) or clonal CD146^Low/High^ MSCs (Low or High) at day 0 (D0) and after 21 days of differentiation (D21). (C) CD34+ haematopoietic stem cells (4 × 10^4^) were cultured on clonal CD146^−/Low^ or CD146^High^ MSCs. At days 7, 14, 21 and 28, non-adherent haematopoietic cells were counted and tested for haematopoietic progenitor content (a). Data are mean ± SEM number of CFU-Granulocytes (b), CFU-Macrophages (c), CFU-Granulocyte Macrophages (d) and BFU-Erythroid (e). Data are mean ± SEM (*n* = 4).

To test whether CD146^−/Low^ and CD146^High^ clones exhibited differences in multilineage differentiation capacity, we tested their ability to differentiate into osteoblasts, adipocytes and chondrocytes *in vitro*. CD146^−/Low^ and CD146^High^ cells did not differ in differentiating into osteoblasts and adipocytes with alizarin red and Nile red staining, respectively (Fig. 2Aa–f). To confirm these data, we quantified osteoblasts by expression of Runt-related transcription factor 2 (Runx2), osterix (Osx), alkaline phosphatase (ALPL) and distal-less homeobox 5 (DLX5); adipocytes by expression of peroxisome proliferator-activated nuclear receptor γ 2 (PPARγ2) and fatty acid binding protein 4 (FABP4); and chondroblasts by expression of as collagen 10 A1 (COLL10A1) differentiation genes by transcriptomic analysis (Fig. 2Ba–g). Results show that CD146^High^ and CD146^Low^ groups did not clearly differ in multidifferentiation capacity. In addition, CD146^High^ and CD146^Low^ cells showed weaker expression of stemness genes (NANOG), octamer-binding transcription factor 4 (OCT4) and SRY-related HMG-box 2 (SOX2) as compared with non-clonal populations (Fig. S2Aa–c). This difference could be explained by the fact that clones underwent more culture during the selection of clones; thus the doubling number was higher for clonal than non-clonal MSCs (20 *versus* 12). Of note, the expression of NANOG and OCT4 was slightly higher for CD146^Low^ than CD146^High^ cells.

Then, to evaluate haematopoiesis support activities *in vitro*, cord-blood CD34^+^ haematopoietic stem cells (HSCs) were co-cultured for 28 days on stroma composed of cells from CD146^−/Low^ or CD146^High^ clones. Non-adherent cells were counted weekly and seeded in multilineage semisolid medium for clonogenic assay. Proliferation of non-adherent CD34+ haematopoietic cells was higher (not significantly) with co-culture on CD146^−/Low^ than CD146^High^ stroma (Fig. 2C a), but total number of CFU-Gs, CFU-Ms, CFU-GMs and BFU-Es were similar on both stromas (Fig. 2Cb–e).

### High expression of CD146 in MSCs is associated with commitment to VSM lineage

**Figure 3:**
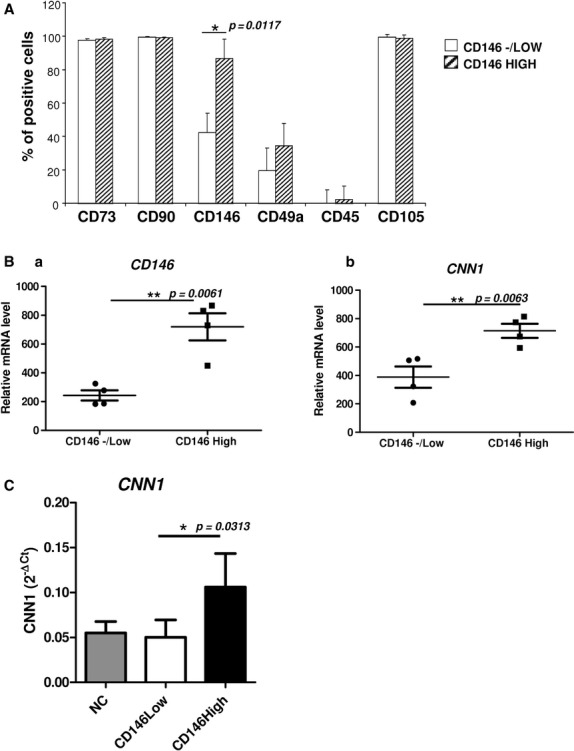
High expression of CD146 in mesenchymal stem cells (MSCs) associated with commitment to vascular smooth muscle cell (VSMC) lineage. (A) Clonal MSCs were phenotyped after expansion. Flow cytometry of fluorochrome-coupled monoclonal antibodies incubated with cells (*n* = 3, *P < 0.05). (B) mRNA expression of CD146 (a) and calponin-1 (CNN1) (b) from gene array data in clonal CD146^−/Low^ and CD146^High^ MSCs. Horizontal bars represent the mean and whiskers represent SEM of four independent experiments. (C) qRT-PCR of CNN1 mRNA level in non-clonal (NC), CD146^−/Low^ and CD146^High^ MSCs.

The levels of the MSC markers CD90, CD73 and CD105 were similar in CD146^−/low^ and CD146^High^ populations derived from clones (Fig. 3A). Interestingly, the expression of CD49a (α1-integrin subunit) was up-regulated in CD146^High^ clones. After their expansion, cells were subjected to gene array analysis. We obtained a list of genes up-regulated in CD146^High^ clones and identified a high expression of CD146, thus confirming our discrimination between studied clones. In this list, we found keratins (KRT7, KRT34, KRT81), CNN1 and LIM-calponin homology domain (LIMCH1) linked to a VSM lineage; insulin-like growth factor binding protein 2 (IGFBP2) expressed in VSMCs; inhibin A (INHBA), a basement membrane protein; and SPARC-related modular calcium binding 1 (SMOC1) and other genes not yet characterized (Fig. 3B, see accession no. GSE48540 in Materials and methods).

Because the CD49a molecule is up-regulated in the VSMC commitment program 20–21, we focalized on VSMC-specific molecules in CD146^−/Low^ and CD146^High^ cells such as CNN1, which was significantly up-regulated in CD146^High^ clonal MSCs (Fig. 3B b). To confirm these data, we performed transcriptome analysis by qRT-PCR of CNN1 and found that CNN1 was significantly up-regulated in CD146^High^ cells (Fig. 3C). We evaluated other VSMC-specific transcripts and found a tendency for up-regulation of early VSMC markers, smooth muscle protein 2α (SM22α) and elastin (ELN) in CD146^High^ cells (Fig. S2B).

**Figure 4:**
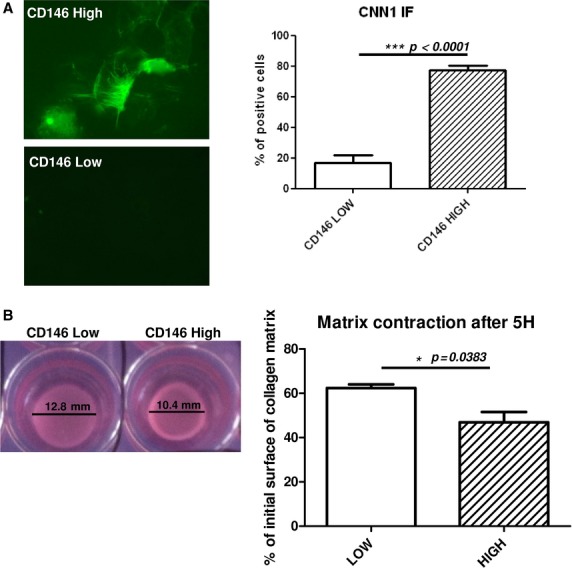
High expression of CD146 in mesenchymal stem cells (MSCs) is associated with vascular smooth muscle cell phenotype and function. (A) Immunoflourescence of CNN1 protein expression in clonal CD146^−/Low^ or CD146^High^ MSCs and quantification. Representative images of four independent experiments. (B) Representative pictures of collagen matrix contraction with diameter value (mm). Collagen contraction assay with clonal CD146^−/Low^ and CD146^High^ MSCs after 5 hrs. Data are percentage of initial surface of collagen matrix (from three independent experiments).

After gene array and transcriptome analysis, we studied CNN1 protein expression in CD146^−/low^ and CD146^High^ populations. Most CD146^High^ clones expressed CNN1 as compared with CD146^Low^ clones (77.3 ± 7% *versus* 16.6 ± 14.7% of CNN1-positive cells, *P* < 0.0001; Fig. 4A). Finally, after VSMC phenotype analysis in clones, we sought to test CD146^High^ and CD146^Low^ subpopulations at a functional level. Thus, cells were assessed for collagen matrix contraction capacity 22–23. Interestingly, CD146^High^ clones contracted more matrix than CD146^Low^ clones at 5 hrs after contraction initiation (51.7 ± 9.4% *versus* 63.3 ± 2.1% of initial surface of matrix; Fig. 4B). Therefore, CD146^High^ MSCs had more VSMC characteristics than CD146^Low^ clones.

### Effect of FGF-2 and TGF-β1 on MSC proliferation, CD146 expression and VSMC commitment

**Figure 5:**
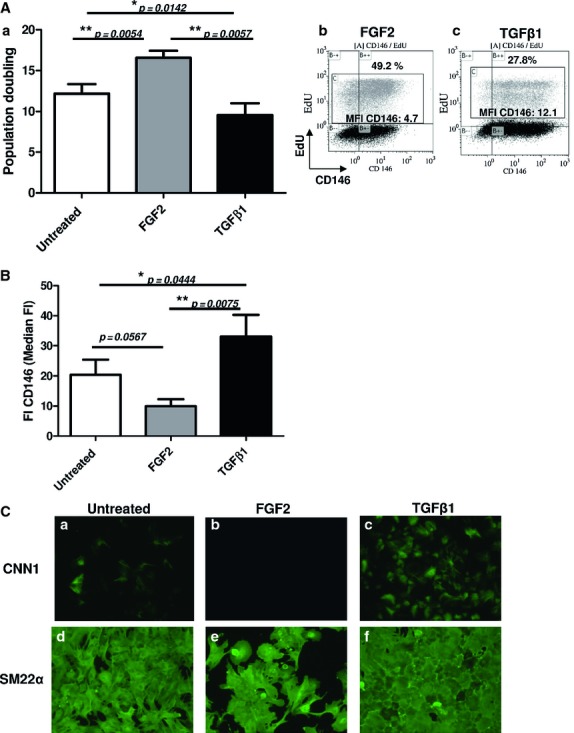
Effect of fibroblast growth factor 2 (FGF2) and transforming growth factor β1 (TGF-β1) on mesenchymal stem cells (MSC) proliferation, CD146 expression and vascular smooth muscle cell commitment. (A) MSCs from crude bone marrow were treated or not with FGF-2 or TGF-β1 for 21 days. Doubling population number was calculated as Log^2^ (cell expansion). Data are mean + SEM from four independent experiments (a). Flow cytometry of MSC population with EdU incorporation in MSCs treated with FGF2 (b) or TGFβ1 (c) from days 3 to 10 of culture. (B) Flow cytometry quantification of CD146 expression (FI) calculated as median fluorescence intensity (MFI) CD146)/isotype control between MSCs treated or not with FGF2 or TGF-β1. (C) Immunofluorescence of protein levels of CNN1 (a–c) and SM22α (d–f) in MSCs treated or not for 10 days with FGF2 or TGFβ1. Images are representative of four independent experiments.

Because TGF-β1 is a key cytokine for inducing VSMC differentiation 16–27, we assessed the effect of TGF-β1 on CD146 expression in MSCs and VSMC differentiation. In addition, we used FGF2, a cytokine with mitogenic effect that promotes proliferation of undifferentiated MSCs *in vitro* 15–28. From crude bone marrow-derived MSCs treated with FGF2 or TGF-β1, we analysed MSC proliferation and CD146 expression over 21 days. First, as compared with untreated cells, FGF2-treated MSCs showed enhanced proliferation (16.6 ± 1.6 *versus* 12.2 ± 2.1 population doubling). In contrast, proliferation was slowed with TGF-β1 (9.5 ± 2.8 population doubling, Fig. 5Aa). Moreover, as determined by flow cytometry with EdU incorporation, the proportion of cycling cells (EdU-incorporated cells) was significantly lower with TGF-β1 than FGF2 treatment (27.8% *versus* 49.2%, Fig. 5Ab and c). Second, CD146 expression was lower in MSCs with than without FGF2 treatment (9.9 ± 3.7 *versus* 20.4 ± 8.4), but higher with TGF-β1 treatment (33.1 ± 12.5, Fig. 5B). In parallel and in agreement with Figure 5B, the median fluorescence intensity (MFI) of CD146 staining on EdU-incorporated cells was up-regulated with TGF-β1 as compared with FGF2 (12.1 *versus* 4.7), so CD146 expression was enhanced by TGF-β1 treatment (Fig. 5A b and c). Furthermore, the presence of a CD146^−^ population after TGF-β1 treatment can be explained by our use of unfractioned BM whole MSC populations in terms of CD146 expression.

To confirm the VSMC commitment by these treated cells, we tested CNN1 expression in all conditions. Untreated MSCs expressed a basal level of CNN1, which was up-regulated by TGF-β1 as compared with FGF-2 (Fig. 5Ca–c). The expression of SM22α, an early VSMC marker, was strong and stable in all conditions (Fig. 5Cd–f). Therefore, similar to the behaviour of single cell-derived CD146^High^ clones, a high level of CD146 expression in the MSC population induced by TGF-β1 was associated with low proliferation and commitment to a VSMC lineage.

## Discussion

Soluble agents as well as cell density, spatial distribution of cells, and solid components of culture (such as extracellular matrix molecules) are increasingly considered critical to cell fate determination and heterogeneity of cultured MSCs. Some experiments suggested that even when derived from a single cell, the progeny of MSCs can be conditioned to behave differently 29–30. This heterogeneity could be a result of extensive *ex vivo* culturing, *in vivo* heterogeneity and plasticity of phenotype reflecting the natural repertoire of MSCs.

To study the different heterogenous subpopulations of MSCs and define specific markers, we focused on CD146, an endothelial and subendothelial marker well described in the MSC literature. CD146, also known as melanoma cell adhesion molecule (Mel-CAM, MCAM) or MUC18 belongs to the immunoglobulin superfamily. In normal adult tissues, CD146 is primarily expressed by vascular endothelium and smooth muscle cells 31,32. Because of the heterogeneity of CD146 expression in MSC populations, we compared CD146^−/Low^ and CD146^High^ cells from clonal and sorted MSCs after culture expansion to determine whether this expression is associated with specific functions. We sorted CD146^High^ and CD146^−/Low^ cells with excellent purity from eight healthy donors after primary culture. CD146^High^ and CD146^−/Low^ cells did not differ in CFU-F frequency, cell expansion or expression of MSC markers (CD90, CD73, CD44, CD105, CD13, CD166) or adherence molecules (CD106, CD49d, CD49f; data not shown). However, this approach has two potential issues: (*i*) the surface re-expression of CD146 in sorted CD146^−^ cells after a short period of culture and (*ii*) the low number of cells obtained after sorting not being amenable to functional studies. To bypass these issues and to have a sufficient quantity of stable CD146− and CD146+ cells to compare, we generated single cell-derived CD146^+^ clonogenic MSCs 7. After expansion of these clonogenic MSCs, most clones expressed CD146 at a moderate level (52% of CD146^int^); 21% were CD146^−/Low^ and 27% were CD146^High^. In this study, we compared clones with extreme levels of CD146 expression, CD146^−/Low^ and CD146^High^.

As previously found, CD146^−/Low^ and CD146^High^ MSCs did not differ in CFU-F number or osteogenic, chondrogenic and adipogenic differentiation capacity 10. Recently, CD146 knockdown in human MSCs was found to disturb proliferation and osteogenic differentiation 34. This disagreement has a few explanations. First, use of RNA interference or ectopic overexpression can have adverse effects on MSC signalling, whereas in our study, we used ‘untouched’ physiological clones expressing different levels of CD146 depending on their fate in culture. Second, we used clones with high population doubling number, whereas the previous authors used MSCs passaged only once.

CD146^High^ and CD146^−/Low^ clones showed similar *in vitro* haematopoietic-supportive activity, which agrees with Tormin *et al*., who demonstrated no difference in stroma-supporting capacity between CD271+/CD45−/CD146^−/Low^ and CD271+/CD45−/CD146^+/High^ fractions in long-term culture initiating cells 10. However, in a xenotransplantation model, contrary to the CD146− fraction, culture-expanded CD146+ cells could re-establish the haematopoietic microenvironment 7. Such discrepancies could be explained by the experimental conditions. We and Tormin *et al*. both used *in vitro* approach whereas Sacchetti *et al*. performed their experiments *in vivo*.

In addition, we found proliferation slightly but significantly higher for CD146^−/Low^ than CD146^High^ MSCs. Accordingly, FGF2 treatment decreased CD146 expression but increased proliferation, as was previously described 7. Conversely, TGF-β1 treatment was associated with increased CD146 expression and VSMC differentiation. The association of CD146 and the VSMC differentiation lineage is reinforced by the fact that the CD146 promoter contains the gene regulation element CarGbox, which is present in all promoters of key genes for the VSMC lineage (αSM-actin, SM22α, CD49a) 33. Alpha1 integrin subunit (CD49a) is one of the SMC-specific marker genes 35. In our study*,* CD49a was up-regulated in CD146^High^ clonal MSCs as was the specific VSMC marker CNN1 at the gene and protein level. In addition, early VSMC markers SM22α and ELN were up-regulated in CD146^High^ MSCs. Also, CD146^High^ MSCs exhibited better potential for contraction of collagen matrix than did CD146^−/Low^ counterparts. Vascular smooth muscle cells are multipotential cells with the ability to differentiate into osteoblasts (resulting in vascular calcification), chondroblasts and adipocytes depending on the inducers (BMP, oxidative stress, *etc*.) 36. Because CD146^High^ MSCs share some properties with VSMCs (*e.g*. phenotype and matrix contraction potential), they would also exhibit multipotentiality. Therefore, CD146 expression by MSCs seems to be a characteristic and predictive marker for commitment towards the VSMC lineage.

Interestingly, the VSMCs were previously related to BM stromal cells with haematopoietic sustaining activities 37. Indeed, the *in vitro* model of long-term haematopoiesis requires a layer of stromal cells generated by Dexter-like culture sytems 38–39. This type of culture constrains BM stromal adherent cells to shift towards the VSMC lineage 38–40. Despite some reports of osteoblastic localization for the HSC niche, others demonstrated a vascular situation 41. In this latter context, HSCs are associated with parasinusal cells, which are similar to advential reticular cells and to parasinusal myoid cells 40,42. All of these cells express αSM-actin. In foetal and adult human BM, αSM-actin-expressing cells form haematopoietic niches 44. Sacchetti *et al*. described CD146+ cells expressing VSMC linage markers (αSM-actin, CNN1, Desmin) with the ability to form a haematopoietic microenvironment 7. Recently, Peault’s group showed in humans that only CD146+ cells could maintain *in vitro* HSCs with long-term repopulating potential 45. Moreover, in agreement with our results, *in vitro* investigations of haematopoiesis-sustaining properties revealed no differences between CD146^+/High^ and CD146^−/Low^ stromal cells; only *in vivo* experiments showed differences. Overall, the CD146^+/High^ MSCs represent a fraction of whole BM-MSCs with few detectable differences as compared with CD146^−/Low^ MSCs. Nevertheless, the differences concerning the commitment towards a VSMC lineage and a high CD146 expression in the MSC population reflect the start of this commitment.

## Supporting information

Figure S1 Sorted CD146^-/Low^ and CD146^High^ MSCs. (A) Crude bone marrow cells were seeded at 5 × 104 cells/cm^2^ in a flask for 21 days. MSCs were sorted into CD146^-/Low^ and CD146^+/High^ cells. (B) CFU-F number from sorted MSCs seeded at 8 cells/cm^2^ in a F25 flask for 10 days. Data are mean ± SEM number of CFU-Fs for 100 cells seeded (n = 8). (C) Population doubling number of non-clonal sorted CD146^-/Low^ and CD146^+/High^ cells after 21 days of culture. Data are mean ± SEM (n = 6).

Figure S2 Expression of stemness genes and VSMC-specific markers by clonal CD146^Low/High^ MSCs. (A) qRT-PCR analysis of mRNA expression of NANOG, OCT4A and SOX2 in non-clonal MSCs (NC), CD146Low and CD146High clones. (B) qRT-PCR analysis of mRNA expression of SM22a and ELN in non-clonal MSCs (NC), CD146^Low^ and CD146^High^ clones. Data are mean ± SEM from three independent experiments.
