# Supplementary figures and images for: CD146 expression on mesenchymal stem cells is associated with their vascular smooth muscle commitment
Source: J Cell Mol Med. 2013 Nov 4;18(1):104–14. doi: 10.1111/jcmm.12168 (PMC3916122; doi:10.1111/jcmm.12168)

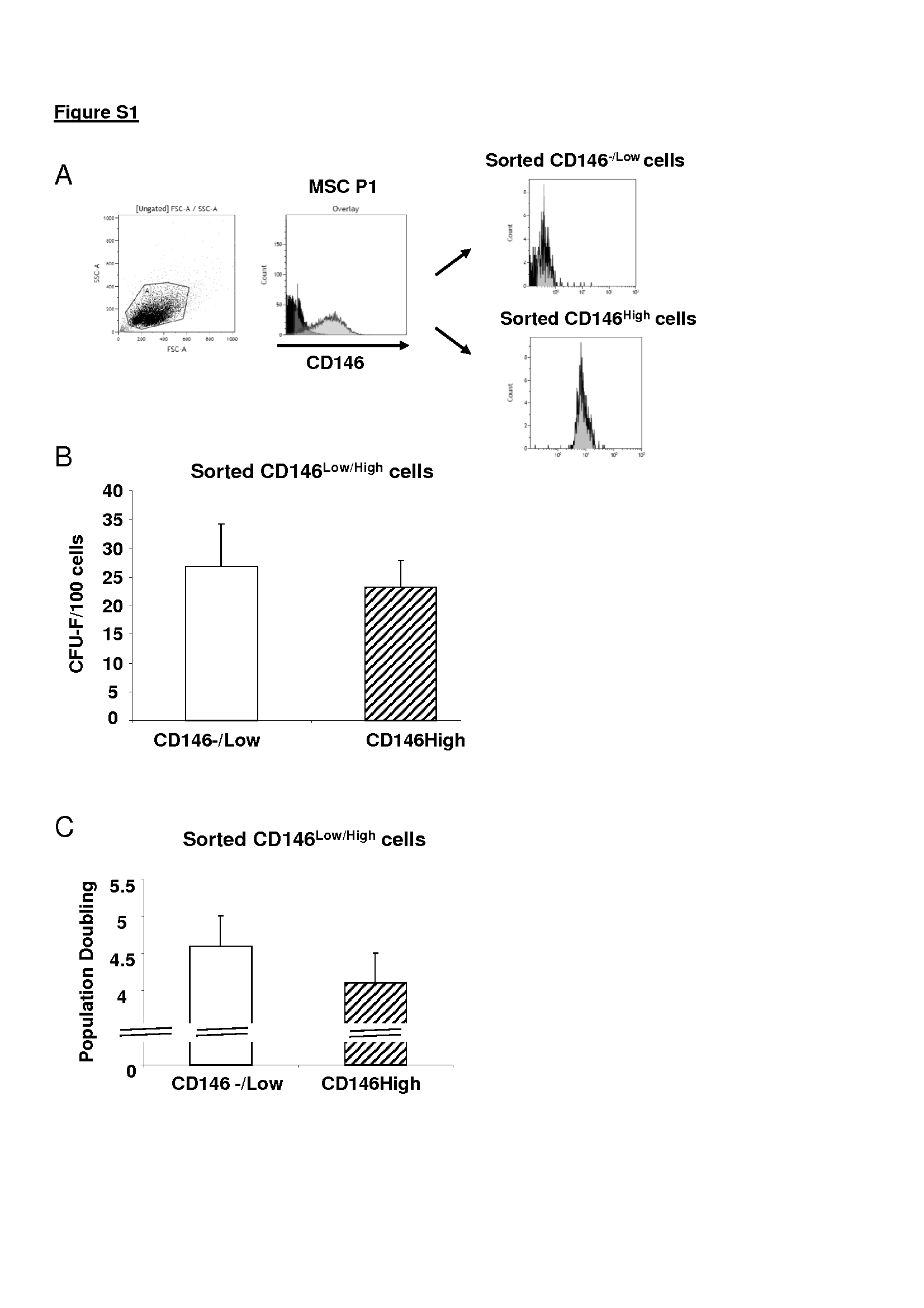

Supplement: Figure S1 — Sorted CD146-/Low and CD146High MSCs. (A) Crude bone marrow cells were seeded at 5 × 10^4^ cells/cm2 in a flask for 21 days. MSCs were sorted into CD146-/Low and CD146+/High cells. (B) CFU-F number from sorted MSCs seeded at 8 cells/cm2 in a F25 flask for 10 days. Data are mean ± SEM number of CFU-Fs for 100 cells seeded (n = 8). (C) Population doubling number of non-clonal sorted CD146-/Low and CD146+/High cells after 21 days of culture. Data are mean ± SEM (n = 6). [file jcmm0018-0104-sd1.tif]

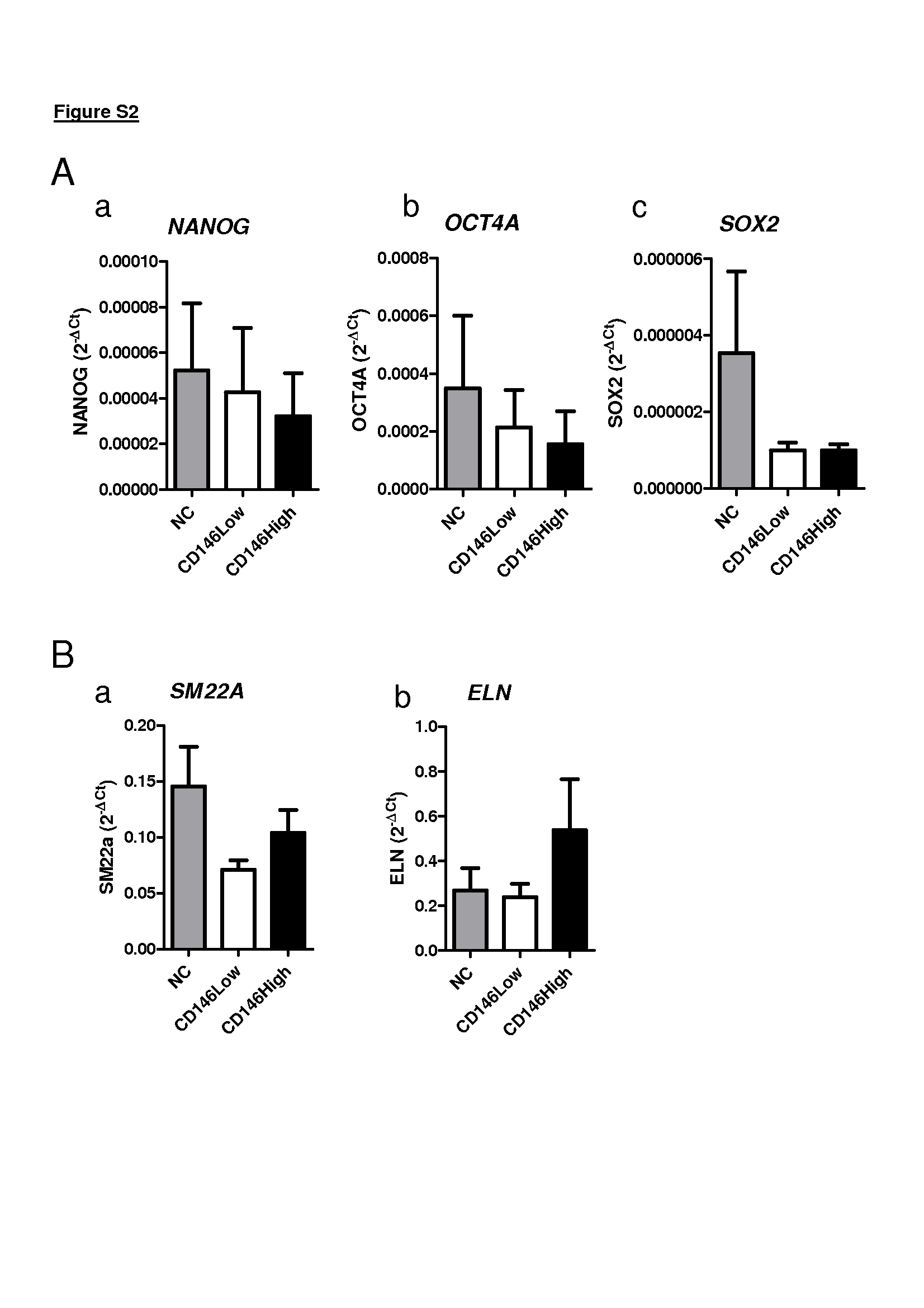

Supplement: Figure S2 — Expression of stemness genes and VSMC-specific markers by clonal CD146Low/High MSCs. (A) qRT-PCR analysis of mRNA expression of NANOG, OCT4A and SOX2 in non-clonal MSCs (NC), CD146Low and CD146High clones. (B) qRT-PCR analysis of mRNA expression of SM22a and ELN in non-clonal MSCs (NC), CD146Low and CD146High clones. Data are mean ± SEM from three independent experiments. [file jcmm0018-0104-sd2.tif]
